# Disaster resilient village-based approach to disaster risk reduction policy in Indonesia: A regulatory analysis

**DOI:** 10.4102/jamba.v13i1.1021

**Published:** 2021-06-23

**Authors:** Saru Arifin, Sonny S. Wicaksono, Slamet Sumarto, Martitah Martitah, Dewi Sulistianingsih

**Affiliations:** 1 Department of Constitutional Law, Faculty of Law, Universitas Negeri Semarang, Central Java, Indonesia; 2 Department of Criminal Law, Universitas Negeri Semarang, Central Java, Indonesia; 3 Department of Civic Education, Universitas Negeri Semarang, Central Java, Indonesia; 4 Department of Private and Commercial Law, Universitas Negeri Semarang, Central Java, Indonesia

**Keywords:** resilience, community, village, disaster risk reduction, preparedness

## Abstract

This article will address the disaster resilience village (DRV) approach as a disaster preparedness method in Indonesia. This scheme became operational in 2012, exactly 5 years after disaster management legislation was passed in 2007. This DRV strategy is a component of the central government’s decentralisation of disaster management to local governments. Using a method of doctrinal legal review, this study argues that the DRV approach to disaster preparedness at the village level is inefficient. That is because the village apparatus is the central player in this DRV, but residents of disaster-prone areas are regarded as an afterthought when it comes to disaster management. Consequently, efforts to strengthen emergency preparedness for residents in disaster-prone areas will be harmed. As a result, it is unsurprising that whenever a disaster occurs in Indonesia, the death toll and damage to property remain high. This is because people who live in disaster-prone areas lack a framework for transforming knowledge and scientific experience with disasters. In addition, this DRV strategy opposes previous disaster experts’ community-based and transformative approaches. However, direct field research on communities living in disaster-prone areas is needed to obtain empirical evidence of the DRV approach’s shortcomings.

## Introduction

Indonesia is covered by three tectonic plates: Indo-Australia, Eurasia and the Pacific. As a result, the country is regularly subjected to severe seismic activity, including earthquakes, volcanic eruptions and other natural hazards (Fahlevi, Indriani & Oktari 2019; Kusumastuti et al. 2014). According to Statista, the tsunami is the most severe threat to Indonesia, with a risk index score of 9.7 out of 10. Meanwhile, drought is the least dangerous hazard, scoring 3.4 out of 10 on the risk scale. In addition, earthquake and flood risk indexes were ranked second and third, respectively, with scores of 8.9 and 8.1, whilst epidemic and tropical Cyclone risk indexes were ranked sixth and seventh, with scores of 6.9 and 6.1, respectively (Statista 2020d).

Meanwhile, natural hazards continue to increase in frequency and severity in Indonesia, both in terms of accidents and fatalities. In 2019, the number of natural hazards increased by 7.2%, whilst the number of fatalities increased by 192% (Dewi 2019). Statista reports that there were 9.392 natural hazards in 2019, up from 2.5 thousand in 2018. Similarly, approximately 5.37 million people sustained personal injuries or were forced to flee their homes as a result of natural hazards, down from approximately 10.2 million in 2018. Around 4.814 people died or went missing as a result of a natural disaster in the same year. Meanwhile, the National Disaster Management Agency (NDMA) recorded three catastrophic events in 2019 that resulted in significant loss of life and property (Statistics South Africa [STATS SA] 2020a, 2020b, 2020c, 2020d).

According to the given data, the probability of catastrophe increases as a result of natural hazards such as earthquakes, floods and landslides caused by heavy rain. Another part of this threat is the community’s continued lack of preparedness for disasters. The underlying problem with this causality is that disaster management, both structural and non-structural, has not been prioritised in regional growth. Efforts to respond to disasters continue to be highly concentrated on disaster emergencies. However, according to the 2005 Hyogo Framework for Action, the government’s responsibility is to protect society from the dangers of disasters and the risks they pose, both in terms of property damage and loss of life (Burkle et al. 2014; Olowu 2010).

Indonesia implemented Disaster Management Law Number 24 in 2007 two years after the Hyogo Framework of Action. Following this Act, the NDMA issued the Disaster Resilient Village (DRV) Guideline in 2012, which serves as a guide for local governments in preparing communities for disaster threats. The DRV is a self-sufficient village that is prepared to react to potential disaster threats and rapidly recover from disaster-related consequences. Regarding these provisions, the Act has been enforced by the majority of local governments through the use of some Regional Legal Products, namely Local Regulations (*Peraturan Daerah*) and Local Head Regulations (*Peraturan Kepala Daerah*). However, the existence of laws, regional regulations and regional head regulations have not been entirely successful in developing new expertise and new cultures within the community to be disaster aware in order to mitigate disaster risk. In many disaster-stricken countries, both human and property deaths continue to be high. This casualty is a product of local governments’ disaster response mindsets, which often prioritise disaster response over disaster preparedness as part of a modern approach to disaster response, namely disaster risk reduction by community capacity building in disaster-prone areas. The primary objective of enacting the Disaster Management Act, which places a premium on human rights in order to protect and secure against natural hazards, is to reduce disaster risk, particularly in regions with a high level of disaster vulnerability (Adnan & Kreibich 2016; Amri et al. 2017).

This article discusses the DRV approach that Indonesia employs in its disaster risk mitigation strategy. The first section serves as an introduction, summarising the article’s overall meaning. The second section will discuss the approaches to disaster risk reduction that academics are currently pursuing. The following analysis will examine the decentralisation of disaster management in Indonesia, from central to local government, followed by an examination of the DRV approach to disaster risk reduction.

## Literature review

When natural hazards occur, humans and their property are the two most fragile objects. As a result, catastrophe experts focus their attention on designing strategies that effectively mitigate the risk of disasters in these two categories. The experts’ commitment to developing effective disaster-reduction strategies began in the 1980s. Numerous studies and international declarations have stated that disaster risk mitigation and the paradigm of community resilience to disasters should be the cornerstones of all countries’ disaster management guidelines (Imperiale & Vanclay 2020).

Numerous researchers have advanced paradigms and techniques for mitigating disaster risk. For instance, a technical approach as a tool for disaster preparedness takes the form of an early warning system (EWS), which is especially useful in the event of a tsunami disaster. Two additional approaches are planning and management. The EWS is geared towards preparing residents living along the river during the flood (Adnan & Kreibich 2016). In addition, some experts argue for a multi-sector or hybrid solution, citing examples such as technology, stakeholders and culture (Karnawati et al. 2011). According to this perspective, the three sectors are inextricably linked in terms of achieving practical disaster risk mitigation outcomes. Disaster risk reduction technology is the product of basic science and technology, and it serves as a repository for massive amounts of disaster data. However, stakeholders’ position in mobilising community resources and capital for disaster risk reduction at the policymaker level is crucial (Mojtahedi & Oo 2014).

In addition, the society’s foundation must be built on disaster risk knowledge and awareness. If these three conditions are met, disaster risk mitigation can be achieved more effectively, according to this strategy (Nahayo et al. 2017). Shohei Matsuura stressed the importance of an interdisciplinary approach in order for today’s experts to successfully develop strategies for disaster risk reduction (Matsuura & Razak 2019). In the light of these divergent approaches, Stefan Hochrainer-Stigler stressed the critical nature of setting global standards to which all countries can adhere to whilst developing community resilience to disasters (Hochrainer-Stigler et al. 2020).

Meanwhile, Douglas Paton introduced a transformative learning strategy centred on disaster-affected populations. It is beneficial to use their experiences during a disaster as teaching material for disaster awareness growth. According to Paton, developing disaster preparedness in disaster-prone areas is challenging if community awareness is not grounded in the perspectives of those affected by disasters. They can gain an understanding of how difficult it is to save oneself during a crisis by drawing on the experiences of disaster survivors. In this regard, the disruptive learning approach to disaster risk reduction appears to be a viable option (Paton & Buergelt 2019).

Given the complexity of the catastrophe problem and existing approaches, the United Nations (UN) announced to the international community an approach that emphasises the critical importance of fully focussing on humanity, which will be directly affected by any disaster. The most recent UN resolution on disaster management includes a human rights approach. Unfortunately, this approach is oriented around a post-disaster scenario, emphasising that humanitarian needs must take precedence over policy considerations during the disaster response process (Lewis & Maguire 2016; Paudel & Regmi 2018).

On the one hand, risk reduction strategies include communities in the recovery process following a catastrophe. According to Newpot and Jawahar, integrating the involvement of communities in disaster-prone areas into a disaster management design is based on clear legal criteria, which is a critical point that contributes positively to disaster management (Newport & Jawahar 2001). This situation demonstrates how the comprehensive disaster management model affects the response to disasters, both by the government and the community, especially in disaster-prone areas.

According to United Nations Development Program (UNDP), disaster preparedness, which is at the heart of the disaster risk reduction model that is currently gaining traction as a new concept and understanding in disaster management in various countries, is futuristic in the sense that it is long-term focussed in disaster management (United Nations Development Program [UNDP] 2001). The disaster preparedness theory seeks to minimise losses caused by disaster threats by introducing adequate countermeasures, maintaining time certainty and maximising organisational efficiency when disasters strike. This definition encompasses all disaster paradigms, whether conventional, modern or most recently established in various parts of the world (Burnham 2006).

In Indonesia, the DRV, also called ‘Desa Tangguh Bencana’, is a novel approach to disaster preparedness. This is in contrast to the approach widely proposed by academics, as discussed previously. Regrettably, this technique has not yet been shown to be effective in dealing with disaster casualties.

## Method

This study took a doctrinal-research approach. The central study focusses on the standards referenced in Indonesian disaster laws – data were gleaned from the national and local legislative texts. Two principles were used to analyse the data: conceptual and regulatory approaches. The data analysis is conducted using a content analysis methodology (Johny 2006; Marzuki 2005).

### The decentralisation of the disaster management

*Article 236 (3) of Acts Number 23 of 2014* on Local Government in Indonesia expressly mentions about decentralisation of disaster management. This Article divides the contents of the Local Government into two main authorities. They are the autonomy scope and the higher rule’s additional power. According to the provisions of the aforementioned Regional Regulation, disaster management is a mandatory function of each local government. The central government’s delegation of disaster management authority to local governments was intended to boost efficiency in Indonesia’s disaster management. As demonstrated by non-uniform disaster data across Indonesian regions, disaster management has been unsuccessful thus far (Subowo 2018). Decentralisation of disaster management policy aims to change bureaucrats’ views of disaster as a multifaceted topic involving social, economic, political and environmental concerns (Mattingly 2002).

In practice, the local government’s disaster management obligations are defined clearly in *Article 5 of Law 24 of 2007*. In the disaster sector, local government affairs are developed through the use of roles and authorities (Article 8 and Article 9). As a result, the local government derives its legislative and administrative powers from the provisions of this disaster management law (Marbun 1997; Syafrudin 2000). The following are the powers expressly delegated to local governments through disaster management legislation:

- the establishment of the Local Disaster Management Agency (LDMA), its functions and responsibilities, organisational structure and legal obligations (Article 18, Article 20, Articles 22–24)
- local governments are responsible for emergency response planning (Article 36)
- assistance and management in the event of a catastrophe (Articles 60–70)
- oversight at all stages of emergency recovery (Articles 71–73).

Several of the powers granted to local governments by the disaster management law are clearly implied, suggesting the law’s purpose and intent despite its general substance (abstract). The local government can issue a new regulation in the form of a decision or a proposal to increase its authority. In addition, the disaster management statute vests local governments with facultative authority. Thus, in this case, the local government has delegated authority to develop alternative policies based on the legal authority established by disaster management legislation (Marbun 1997).

The presence of legal standards governing local governments’ functions and authority in disaster management has implications for the diversity of their responses in implementing disaster regulation policies in their respective areas, both in defining their local duties and authorities and in planning, funding and supervising disaster management.

Meanwhile, Gerber and Robinson proposed various forms of ‘decentralisation’ for disaster management, especially for disaster preparedness. Firstly, the term refers to certain geographical areas within certain local government jurisdictions. Secondly, decentralisation led to the collaboration between federal and regional governments in order to mitigate the risk of catastrophic disasters. Thirdly, decentralisation refers to the ‘substance of collaborative problem solving’ when it comes to resolving problems in government without the use of formal administrative boundaries. According to Gerber and Robinson’s report, Indonesia appears to be similar to the first category in terms of ‘decentralisation’ of disaster management. The first category has been used in disaster management activities where regions are not adequately prepared for disasters but yet include the centre in preparation and mitigation (Gerber & Robinson 2009).

#### Local government’s responsibility and authority in disaster management

According to *Article 8 of Law No. 24 of 2007* on disaster management, local governments have four responsibilities in disaster management implementation, namely

- to ensure that disaster-affected people and refugees have their rights respected in compliance with minimum service standards
- to protect the community from disaster-related effects
- disaster risk reduction and the integration of disaster risk reduction into growth programmes
- appropriate and adequate funding for emergency response in the regional budget.

The provisions of the Article categorise the local government’s disaster management responsibilities into two categories: response and preparedness. It ensures that people’s rights are respected and that disaster-affected refugees are trained in response policy. Meanwhile, emergency preparedness is being introduced to safeguard the populace from disaster-related consequences. It entails aligning disaster risk reduction strategies with development programmes and allocating appropriate funds for disaster recovery within a sound Local Revenue and Expenditure Budget.

Meanwhile, *Section Chapter II of the Regulation of the Head of NDMA Number 3 of 2008* on the Guidelines for the Establishment of the LDMA outlines four obligations of the local government to protect the public from the danger and impact of disasters, including the following:

- providing information and raising awareness about disaster hazards and risks in the region
- providing educational opportunities about disaster hazards and risks
- providing financial assistance
- providing social protection and security, especially for vulnerable groups
- mitigation, preparedness, emergency response, rehabilitation and reconstruction.

Numerous local government leaders emphasise that disaster recovery is a strictly local responsibility that is entirely dependent on community resilience. As a result of the central government’s emphasis on community resilience in the face of disasters, it offers policy guidelines that supplement the minimum standards established by local governments. Minimum service standards are regulated by *Government Regulation Number 2 of 2018* concerning Minimum Service Standards. Article 9, paragraph (3) highlights points b, c, d and e, as follows:

- disaster-prone information systems
- disaster prevention and preparedness programmes
- emergency and evacuation services for disaster victims
- rescue and evacuation services for fire victims.

The provision demonstrates that the disaster management character regulated by the disaster management law, which is primarily related to a local government authority, continues to rely on a traditional approach. It excludes the conventional disaster management cycle, which encompasses preparedness, response, recovery, and mitigation, since it ensures that emergency response is prioritised in the event of a disaster. The second party, preparedness, on the other hand, shows the strategy’s modernity (Mattingly 2002; UNDP 1992). As a result of Asia’s regional scale, models of traditional disaster response approach exist in every country and culture, as Mattingly stated:

The traditional approach has been to wait until a disaster occurs, then work nights and day to deal with the aftermath. Some societal and religious beliefs foster a fatalistic attitude toward disasters as expressions of ‘the will of God’. However, [as opposite] current trends throughout Asia and the world include: focusing more emphasis on pre-disaster mitigation, linking mitigation with ongoing development activities, and forming partnerships between national and local government and non-governmental organizations to promote risk reduction and disaster preparedness. (Mattingly 2002:19)

According to Mattingly’s remark, one of the goals of the 2007 passage of Law Number 24 is to alter the conventional approach to disaster management whilst also eroding traditional beliefs about disasters as simply God’s ‘willing’ that humans must accept. As a result, the Disaster Management Law’s spirit is to encourage a move away from fanatical and theological views on disaster problems and towards rational and analytical thinking. This new mindset would radically alter traditional society’s response to crises, preparing it to deal with them objectively and methodically rather than giving up. In addition, this concept modifies the mindset of ‘passive religion’ to make it more rational, especially in terms of comprehending God’s destiny. The person who has been endowed by God with the gift of reason must use it to seek solutions to the problems they face, rather than simply giving up (Pujiono 2007).

In contrast to the local government’s view of its responsibilities that places a greater emphasis on response than on preparedness; Article 9 paragraph (3) of the Government Regulation on Minimum Standards of Services and *Article 9 of the Disaster Management Law* follow a more structured cycle, stressing the following aspects of preparedness:

- designing disaster management policies that are consistent with local planning policies
- developing growth plans that incorporate elements of disaster management policies
- establishing strategies for emergency management cooperation with other provinces or districts
- regulating local government’s use of technology as a source of threat or danger from disasters
- the formulation of policies to guard against the mismanagement and degradation of natural resources in excess of nature’s capacity in the area
- regulating the collection and distribution of funds or products at the regional, district or city level.

A study of several provincial regulations in Java, Sumatra, Sulawesi, Kalimantan and Nusa Tenggara shows that whilst several local regulations share significant similarities, their enactment dates differ significantly. However, these local regulations were adopted under the 2007 Disaster Management Legislation. This legislation explains how municipal laws were enacted in various provinces to carry out disaster management law provisions. The LDMA places those agencies in charge of disaster management, which includes preparedness and prevention, emergency response and recovery. Certain local laws also have similarities in terms of the variety of operations conducted at each stage of disaster management, as shown (Table 1).

**TABLE 1:** Disaster management in the local regulation.

Disaster management stages	Variable
Preparedness and mitigation	There are two policies in the pre-disaster: in the no disaster situation, in the prone-disaster situation (preparedness, early warning system and mitigation)
Response	Consisting of six activities: quick detection of all damaged housing and building determination of the disaster status evacuation and mitigation of the victims providing basic needs protecting of the vulnerable groups rehabilitation of the shelters.
Recovery	Consisting of two activities: Rehabilitation: ▪ maintaining the destruction of the environment ▪ maintaining the public utilities ▪ the assistance of the shelter reconstruction ▪ recovery of mental and psychological of the victims ▪ healthy services ▪ reconciliation and reintegration of social conflict ▪ recovery of social, cultural and economics ▪ recovery of social security ▪ recovery of the government activities ▪ recovery of the public services. Reconstruction: ▪ reconstruction of the public utilities ▪ reconstruction of the social facilities ▪ reconstruction of the social economy ▪ application of the disaster-resistant building ▪ public participation and business ▪ development of the social, economy and cultural ▪ increase of the public services.

As shown (Table 1), the disaster management system is monitored at all levels, including contact and command lines. This stage demonstrates compliance with the Disaster Management Law and Regional Government Law Number 23 of 2014, emphasising the Local’s authority. In addition, various local government emergency response statutes integrate technology as a research product. This, however, is limited to the use of disaster EWS. In contrast to developed countries such as Japan, disaster management systems integrate science into every aspect of development, infrastructure and education (Mattingly 2002).

#### Disaster preparedness concept design

A study of some local laws on disaster management is divided into two categories: those that apply in the absence of a disaster and those that apply in the presence of a disaster. Without regard for natural hazards, the municipal government enacts the following policies:

- risk mitigation in the event of an emergency
- disaster recovery planning
- prevention
- integration into the planning of future growth
- the framework for assessing disaster risk
- implementation and adherence to spatial plans education and training
- quality standards for professional emergency management.

Local codes define a broad spectrum of emergency preparedness practices that are very extensive conceptually. Local governments, for example, integrate it into their 5-year Local Development Plan (LDP), which is updated every 2 years, as part of their emergency response plans. This policy is consistent with the 2008 Hyogo Framework of Action, which place a premium on disaster prevention and preparedness as critical components of the government’s development policies.

Each local government has a unique set of activities in their numerous Local Medium-Term Development Plans (LMTDs) documents to enforce disaster risk reduction by preparedness activities. For example, Central Java’s policy choice [via *Local Regulation No. 5 of 2019*] is focussed on a social and technical approach to disaster management. The government of Central Java establishes DRV for social policy. It is hoped that by establishing the DRV, communities in disaster-prone areas will be able to react and cope independently to threats, as well as quickly recover from disaster-related effects. Several critical DRV indicators include the following (Bappeda 2018a):

- enhancing the efficiency of critical services
- enhancing catastrophe risk management
- integrating climate change adaptation into community empowerment processes
- coordinating disaster risk management services, enhancing emergency preparedness processes
- recovering and reconstructing after disaster.

According to the Central Java Local Development Agency, at least 1674 villages, or 19.5% of all villages in Central Java, were considered flood-prone, whilst 2136 villages, or 24.9%, were considered landslide-prone. Between 2014 and 2019, the number of DRVs increased from 34 to 68 (Bappeda 2018a).

As part of another programme, Central Java also installed an EWS in disaster-prone areas. As a result of the high cost of this EWS technology, the RDMA of Central Java and the Ministry of Energy and Mineral Resources are collaborating on the procurement. Between 2014 and 2018, 46 EWS were deployed, with RDMA providing details on 17 EWS and the Ministry of Energy and Mineral Resources providing details on 29 EWS. As a result, Central Java’s preparedness efforts are focussed on creating DRV that prioritises community participation. East Java Province, such as Central Java, takes a collective approach to preparedness by establishing DRV. This software tends to be a cornerstone of the government of East Java. This initiative was deemed necessary because of the DRV’s rapid growth in the province’s districts. There were 14 resilient villages in 2014, but there were 284 by the end of 2018.

Unlike the Central and East Java Regional Governments, the West Java Regional Government implements a preparedness policy by strengthening the Disaster Risk Index database to support disaster threat reduction programmes. In addition, the alternative policy type is strengthening human resources and emergency response agencies. Creating a Disaster-Resilient School is an example of a tangible human capital strengthening activity (Bappeda 2018b).

Other provinces, including Aceh, West Sumatra, West Kalimantan, South Sulawesi and West Nusa Tenggara have adopted the three Java provinces’ disaster preparedness strategies. Their policy in disaster-prone areas is focussed on community preparedness and the use of EWS technology (Bappeda 2016, 2017, 2019). However, most regions favour the DRV approach as a policy instrument for disaster preparedness. According to this strategy, DRVs formed on average between 2016 and 2020 remain operational. This process is inextricably linked to the NDMA-mandated uniformity of approaches.

West Nusa Tenggara took a slightly different approach to developing disaster preparedness policies by establishing the Disaster-Resilient City (DRC). This is an interesting idea because it contrasts with the NDMA’s description of disaster management decentralisation, which focusses exclusively on village communities at the lowest government level. The government of West Nusa Tenggara’s priority is to educate the DRC about how vulnerable cities are to disasters. Local preparation is incompatible with disaster risk mitigation, which is mostly used for emergency response strategies such as mobilising aid and rescuing victims. With frequent traffic congestion in major cities such as Jakarta, Surabaya and Bandung, it is easy to imagine how, in the event of a disaster, assistance and victim rescue can be carried out quickly. This is why the DRC’s community resilience planning strategies must demonstrate dedication and flexibility (Norris 2002).

### The disaster resilience village approach

Public participation, as a preferred form of community preparedness for local governments, is critical in fostering disaster risk reduction strategies aimed at minimising casualties and damage (McEntire & Myers 2004; Paton 2003). This policy assistance is consistent with the views of disaster specialists, who stress the ineffectiveness of disaster preparedness in the absence of disaster-prone populations. On the other hand, strengthening their capacity to engage effectively in disaster risk reduction planning will improve their confidence and resilience in the face of disasters (Newport & Jawahar 2001).

In addition, community engagement in disaster preparedness is crucial because individual involvement from disaster-prone communities is insufficient; community participation, aided by non-governmental organisations and government agencies, is needed. When a disaster occurs, community involvement will assist residents in identifying their available resources, expertise and adaptations for emergency response. As a consequence, community involvement in disaster preparedness is a social mechanism that enables disaster-prone communities to organise themselves in the event of a disaster that necessitates self-rescue and the use of all available resources (Newport & Jawahar 2001). Gilbert continued by stating that the response to disasters was to spread [affected people’s] needs and to build their resilience in anticipation of possible disasters (Burnham 2006, 2011).

The strategy for involving the community in disaster preparedness is divided into four stages: strategic planning, community readiness, task forces and a disaster response mechanism (Newport & Jawahar 2001). The party is active in village-level disaster planning. In practice, societies organise a range of activities around which villagers must agree in order to save lives, shelter and property during a disaster. In addition, village-level preparedness is carried out by involving a large number of villagers in order to increase disaster awareness. In this regard, the population was divided into several groups, including women, farmers and others. The task force places a stronger emphasis on groups of young people and women allocated community roles, such as 1:10, in which one person is responsible for 10 family members. This, however, is based on the population density of the village. This task force group is responsible for everything that occurs prior to, after and after the disaster. As a result, they must be educated in crisis management’s three stages (Newport & Jawahar 2001).

These community participation criteria are interpreted as DRVs or resilient towns in disaster preparedness management in the regions. These standards place a premium on populations living in disaster-prone areas’ intense readiness to become acquainted with their environment and to enhance their ability to cope with disaster situations. This capability developed as a result of comprehensive disaster management planning, which included the inclusion of disaster data and information as part of the EWS. As a consequence, Chapter II of the Regulation of the Head of NDMA Number 1 in 2012 Concerning General Guidelines for DRVs emphasises the following components that [must] be accessible in DRVs:

- village law governs disaster risk reduction and management. The village regulation’s content, as autonomous rule, reflects the local village community’s agreement to be guided and embraced collectively by the entire village community
- the approach is a compilation of village disaster preparedness plans, contingency plans and village development plans
- the entity creates a village emergency management forum, which is comprised of representatives from all village government, culture and disaster management volunteer groups, including neighbourhood associations (RT and RW). In addition, this forum facilitated cooperation between industries and stakeholders to advance disaster risk reduction efforts
- funding is the distribution of funds specifically designated for disaster relief in the village
- capacity building through training, education and knowledge sharing with the community, particularly voluntary organisations and emergency management actors, to enable them to prepare for, implement and evaluate disaster risk reduction measures.

By implementing disaster management programmes, such as human, structural and non-physical mitigation activities, an EWS, emergency response readiness and all efforts to reduce disaster risk through construction interventions and non-structural recovery facilities are developed. According to the Regulation of the Head of NDMA, the formation and compilation of the DRV programme shall conform to the Hyogo Framework for Action, which covers the most recent disaster management cycle preparedness, prevention, response and recovery. As a result, the NDMA provides several criteria for DRVs and classifies them into three groups based on determined assessment scores: main, intermediate and primary. On the other hand, the ranking’s scale has no valid reason other than the fact that it is the set’s only classification. This classification is distinct from estimating disaster vulnerability using formulas adapted from the international context in order to make the rational explanation more readily understandable. The criteria and scores for the three groups of DRVs are given (Table 2).

**TABLE 2:** Types and parameters of disaster-resilient villages.

Number	Types of DRV	Criteria	Score
1	Primary	having a DRR policy outlined in village regulations having disaster management planning documents as outlined in the Village Medium Term Development Plan and detailed in the Village Development Activity Plan having a DRR forum consisting of community representatives, including women and vulnerable groups and village government representatives having a volunteer team of disaster management having a system of disaster risk assessment, risk management and vulnerability reduction, including productive economic activities to reduce the risk of vulnerability having a system to increase disaster preparedness and response capacity	51–60
2	Middle	a draft DRR policy developed having disaster management planning documents but not integrated into the village medium-term development plan having a DRR forum but not yet active having a volunteer team of disaster management, but not yet fully functional and active disaster risk assessment and disaster management systems already exist but have not yet been tested for reliability capacity-building efforts	36–50
3	Low	have an initial initiative to develop DRR policy efforts to draw up DRR planning documents an effort made to form a village volunteer team an effort made to form a DRR forum initiatives conducting disaster risk assessments and risk management and vulnerability reduction are in place initial efforts to increase disaster preparedness and response capacity	20–35

DRV, disaster resilience village; DRR, disaster risk reduction.

According to NDMA, the administrative method used to plan the DRV clusterisation is intended to serve as a model for DRV growth. The NDMA acknowledges that some parameters for assessing DRV clusters remain subjective and simple. As a result, more objective instrumentation for evaluation will be created. The criteria and clustering of DRVs facilitate the government’s provision of necessary policy interventions in this context. It should be remembered, however, that the cluster of DRVs should not be the subject of public concern, much less on the ‘political economy and disaster issues’ related to the budget allocated. A crucial concern that all stakeholders interested in rural community growth must resolve is their resilience in the face of disaster risks.

Based on the addressing point stated above, NDMA stresses that the activities carried out in the DRV include four things:

- Compilation of village maps and plans
  ▪ a village risk map, countermeasures plan and village action plan
  ▪ map of evacuation routes and places of refuge
  ▪ community action plan
  ▪ community-based EWS
- Formation of village volunteers
  ▪ twenty people per village
  ▪ expertise and skills include: SAR, first aid, logistics, public kitchen and communication
- Community training
  ▪ training for village officials
  ▪ training for villagers
  ▪ volunteerism training
  ▪ planning training
- Compilation of village legislation
  ▪ compilation of village regulations
  ▪ formulation of village head regulations
  ▪ preparation of decree of the village head.

This recommendation tends to be prudent and methodical. However, putting the advice into action is not always straightforward. Two critical problems in the village are human resources and utilities. As a result, all stakeholders in the village’s disaster risk reduction efforts must take this situation seriously. Gerber and Robinson claimed in relation to this issue that the documentary approach to assessing disaster preparedness has two weaknesses. Firstly, it is costly and time-consuming, particularly when the community is evaluated on a large and broad scale. Secondly, when it comes to disaster preparedness, the emphasis is always on the ‘document context’, rather than on whether the information contained in the assessment document accurately reflects the real disaster preparedness circumstances (which is not always the case) (Gerber & Robinson 2009).

According to the village disaster-resilient guidance, disaster risk reduction efforts should be directed primarily at village apparatuses, not at the population living in disaster-prone areas. From this vantage point, it is reasonable to assume that this proposal was not implemented adequately. As a result, they receive only what the village emergency team directs and all services are developed in accordance with their view of the disaster threat, rather than the community’s experiences and feelings. This condition will mean that the grass-roots approach to disaster risk reduction has been successful.

In addition, the translation of the DRV approach to village elites as competent parties rather than communities living in disaster areas in terms of implementing disaster preparedness policies is inconsistent with the provisions of *Article 8 of Law No. 24 of 2007* on Disaster Management, which directs the Regional Government to implement disaster risk reduction policies for communitarian purposes. This is endorsed by *Article 9 (3 point b) of Government Regulation No. 2 of 2008* on Minimum Service Standards, which directs (Local) governments to implement disaster risk reduction and disaster preparedness strategies (for communities susceptible to disasters).

In addition, the policy runs counter to the scholar’s recommendation that the community acts as the front line for disaster preparedness. As community involvement would recognise their resources, capabilities and adaptation to the mechanism of action (in the event of a disaster), by involving them in emergency preparation, disaster-prone communities will organise themselves in the event of a disaster that necessitates self-rescue and the use of all available resources (Kusumastuti et al. 2014; Newport & Jawahar 2001; Paton & Buergelt 2019).

## Conclusion

The DRV approach to disaster risk reduction in Indonesia conceptualises the role of village elites in ensuring community disaster preparedness. On the other hand, civilians in disaster-prone areas are the first concern of a disaster response team. This policy runs counter to the provisions of *Article 8 of the Disaster Management Act 2007* and *Article 9 of the Government Regulation* 2008 on the Minimum Service Standards for Disaster Management, which both state that the population living in disaster-prone areas, not village elites, should be specifically prepared to face disaster danger. Furthermore, this approach runs counter to the scholar’s suggestion that communities place a premium on disaster preparedness. Consequently, without a plan in place, on-the-ground disaster risk reduction efforts may fail. It is evident from this vantage point that Indonesian society continues to be vulnerable to disaster threats and vulnerabilities.

However, as this study is primarily concerned with regulatory issues, additional empirical evidence from various disaster-prone communities in Indonesia is needed to support this conclusion. According to this study, decentralised disaster management should allow each region to develop multiple approaches to disaster risk reduction that are tailored to the characteristics of the disaster-prone area. In addition, each solution should be grounded in empirical evidence from the vulnerable area. As a consequence, the previous catastrophe’s transformative lesson is intended to extend to residents of disaster-prone areas who are contemplating how to protect their lives and property in the event of a disaster.
